# *Fusobacterium nucleatum* Aggravates the Progression of Colitis by Regulating M1 Macrophage Polarization via AKT2 Pathway

**DOI:** 10.3389/fimmu.2019.01324

**Published:** 2019-06-12

**Authors:** Le Liu, Liping Liang, Huifen Liang, Mingming Wang, Bingyun Lu, Meng Xue, Jun Deng, Ye Chen

**Affiliations:** Guangdong Provincial Key Laboratory of Gastroenterology, State Key Laboratory of Organ Failure Research, Department of Gastroenterology, Nanfang Hospital, Southern Medical University, Guangzhou, China

**Keywords:** *Fusobacterium nucleatum*, ulcerative colitis, gut microbiota, M1 macrophage, mucosal barrier

## Abstract

Disordered intestinal flora and discordant immune response are associated with the development of ulcerative colitis (UC). Recent work has described the ability of macrophages to undergo repolarization toward a proinflammatory M1 or anti-inflammatory M2 phenotype in response to particular bacterium-derived signals. *Fusobacterium nucleatum* (*F. nucleatum, Fn*) is a species of intestinal commensal bacteria with potential pathogenicity, but its association with UC and how it may contribute to progression of UC is largely unknown. In this study, we provide new evidence that *F. nucleatum* accumulated heavily in the intestine of UC patients and was accompanied by the secretion of IFN-γ and the skewing of M1 macrophages. Mechanistically, our data showed that *F. nucleatum* aggravated dextran sodium sulfate (DSS)-induced colitis in the production of Th1-related cytokines IFN-γ through the AKT2 signaling pathway *in vitro* and *in vivo*. To further confirm the disease-relevance of these shifts in macrophage repolarization in response to *F. nucleatum*, stimulated bone marrow-derived macrophages (BMDMs) were transferred into recipient mice with DSS colitis. This transfer resulted in increased disease activity and inflammatory cytokine production. Taken together, we show clearly that *F. nucleatum* can promote the progression of UC via proinflammatory M1 macrophage skewing, and targeting *F. nucleatum* or AKT2 signaling may be a viable means of blocking development of UC.

## Introduction

The mucosal tissues are densely colonized with myriad bacterial species that together form a commensal microbiome. As these tissues are often the initial source of pathogen contact, local immune cells including macrophages regularly sample intestinal materials to detect problematic microbes (1–3). Once activated, these macrophages can both act to kill microbial pathogens and to trigger a local or systemic immune response (4, 5). These macrophages are able to detect and respond to pathogens via pattern recognition receptors (PRR), which recognize particular microbe-associated molecular patterns, such as lipopolysaccharides (LPS), lipoteichoicacids (LTA), or particular nucleic acid ligands. PRR-mediated macrophage activation enhances cellular activation and phagocytic activity (6, 7).

Many studies have revealed that macrophages are relatively plastic, and that the phenotype they adopt can markedly affect local mucosal innate and adaptive immune responses (8, 9). Traditionally, macrophages are divided into two major classes: the classical proinflammatory M1 class, and the alternatively activated immunomodulatory M2 class (10). Both the local tissue microenvironment and the microbes being detected by macrophages can influence the resultant macrophage differentiation, activating distinct signaling pathways in a context-dependent manner (11, 12). Classically activated M1 macrophages adopt an inflammatory phenotype, enhancing cytotoxicity, localized fibrosis, and tissue damage in response to autocrine or paracrine IFN-γ secretion (13, 14). In contrast, macrophages with an M2 phenotypes develop in response to autocrine or paracrine IL-4 and IL-13, and promote tissue repair and enhance local cell regeneration (15). Additionally, inactive macrophages can develop in response to IL-10, acting primarily as phagocytic cells involved in remodeling the local tissue environment (16).

Ulcerative colitis (UC) is a disease of the colon and rectum, typically affecting the innermost lining mucosa and leading to the formation of inflamed regions lacking normal mucosal morphology (17). The disease is characterized by chronic inflammation with a relapsing and remitting course arising from a complex series of environmental, genetic, and immune features. The local dysfunctional intestinal flora and their products are believed to play a key role in the development of inflammatory bowel disease (IBD), which includes UC as well as the related Crohn's disease (18–20). Meanwhile, abnormal immune responses to symbiotic bacteria can lead to the formation of intestinal mucosal lesions, including extended epithelial damage, immune cell infiltration, crypt abscesses, and chronic inflammation, all of which are characteristic of UC. Although there is evidence to support this conclusion, the extensive diversity of the gut microbiome makes it difficult to precisely determine whether specific microbes are associated with UC development or progression. Even so, recent work has provided evidence that certain invasive species of *Escherichia coli (E.coli)* and *Fusobacterium* are promising candidates for such an association (21).

There is extensive heterogeneity among *Fusobacterium* species, with some acting as opportunistic pathogens that can drive inflammatory diseases such as periodontitis or appendicitis in particular contexts (22, 23). Recent work has revealed that *Fusobacterium nucleatum* (*F. nucleatum, Fn*) accumulated heavily in colorectal cancer (CRC) and is closely related to the drug resistance of chemotherapeutic drugs (24–26). It is well known that excessive chronic inflammation is a key risk factor for tumor formation, and some studies have found that *F. nucleatum* isolated from biopsy tissue samples originating from IBD patients was more invasive than those isolated from healthy tissues (24, 27), so we speculated that *F. nucleatum* may be an important driver of the progression of IBD and the formation of CRC. However, the relationship between *F. nucleatum* and inflammation and how *F. nucleatum* acts in the pathogenesis of IBD, have yet to be determined.

In this present study, we investigated the link between *F. nucleatum* and M1 macrophage polarization in the context of colitis development, and further elucidate the regulation mechanism between them. Our findings suggest that *F. nucleatum*-mediated M1 macrophage skewing may be a key pathway driving inflammatory tissue damage in DSS-colitis in response to Th1 cytokines.

## Results

### *F. nucleatum* Is Present and Associated With Altered Macrophage Polarization in UC Patients

**Figure 1:**
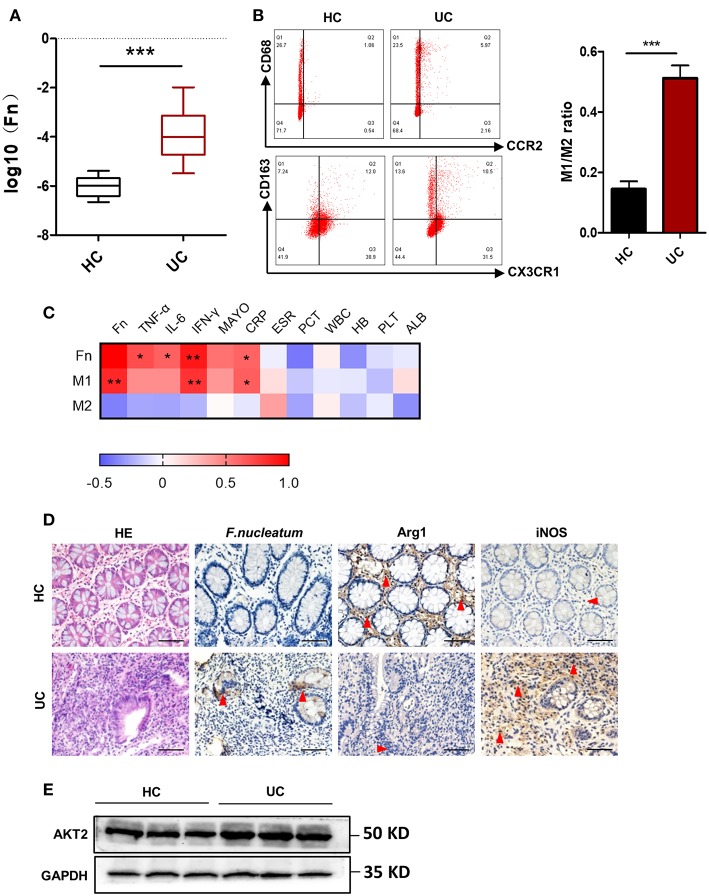
*F. nucleatum* is present and associated with altered macrophage polarization in UC patients. **(A)** Absolute fluorescence quantification of fecal *F. nucleatum* in 30 UC patients and 16 controls. **(B)** M1 (CD68+CCR2+) and M2 (CD206+CX3CR1+) populations in peripheral blood from those with UC (*n* = 12) and controls (*n* = 12), as measured by flow cytometry. The difference of M1/M2 ratio between two groups was analyzed. **(C)** Correlations between *F. nucleatum* abundance, inflammatory cytokines and clinical parameters from 12 active UC patients. Media values were correlated, with a heatmap of Spearman's *R*-values. + indicate the Kendall rank correlation. Black margins denote those correlations that were significant even when analyzing individual samples based on Kendall's *p*-values. **(D)** Hematoxylin and eosin (H&E) and immunochemistry (IHC) staining for *F. nucleatum*, Arginase 1 (Arg1, M2 specific marker), and inducible nitric oxide synthase (iNOS, M1 specific marker) in sections of colon tissue from UC patients (*n* = 12) or healthy controls (*n* = 12). Scale bars, 25 μm. **(E)** Western blot analysis of AKT2 expression in UC patients (*n* = 12) and healthy controls (*n* = 12). Data are presented as means ± SD. ^*^P < 0.05; ^**^P < 0.01; ^***^P < 0.001; Student's *t*-test (two-tailed). CRP, C-reactive protein C; ESR, erythrocyte sedimentation rate; PCT, procalcitonin; WBC, white blood cell; HB, hemoglobin; PLT, platelet; ALB, albumin.

*F. nucleatum* has been shown to be present at high levels in those with IBD, and recent work suggests that the pathogenesis of this disease is linked with an excessive inflammatory response to normally present bacteria in susceptible individuals (28). We therefore sought to measure the presence of *F. nucleatum* in those with UC via absolute fluorescence quantification based on 16s rDNA measurement. We found that levels of these bacteria were significantly higher in fecal samples of patients with UC relative to healthy controls (Figure 1A). Given their important status as immunoregulatory cells, we next investigated the link between macrophages and *F. nucleatum*. By analyzing peripheral blood samples from healthy controls (HC) and UC patients, we found increased M1 macrophage levels both intestinally and peripherally in those with UC (Figures 1B,D). Comparing these *F. nucleatum* levels with other available common clinical indicators of UC, we found a positive correlation between microbe load, M1 macrophages, Mayo score, CRP, and IFN-γ levels (Figure 1C). These data highlight a potential role for *F. nucleatum* in UC progression. Besides, AKT2 plays an important role in the formation of chronic inflammation and macrophage differentiation. Therefore, we detected the protein expression of AKT2 in UC patients and HC. The results showed that the expression of AKT2 in UC was significantly higher than that in the HC (Figure 1E). This suggested that AKT2 may be involved in the regulation of macrophage polarization by *F. nucleatum* in the context of UC.

### *F. nucleatum* Increased the Susceptibility of Mice to DSS-Induced Experimental Colitis

**Figure 2:**
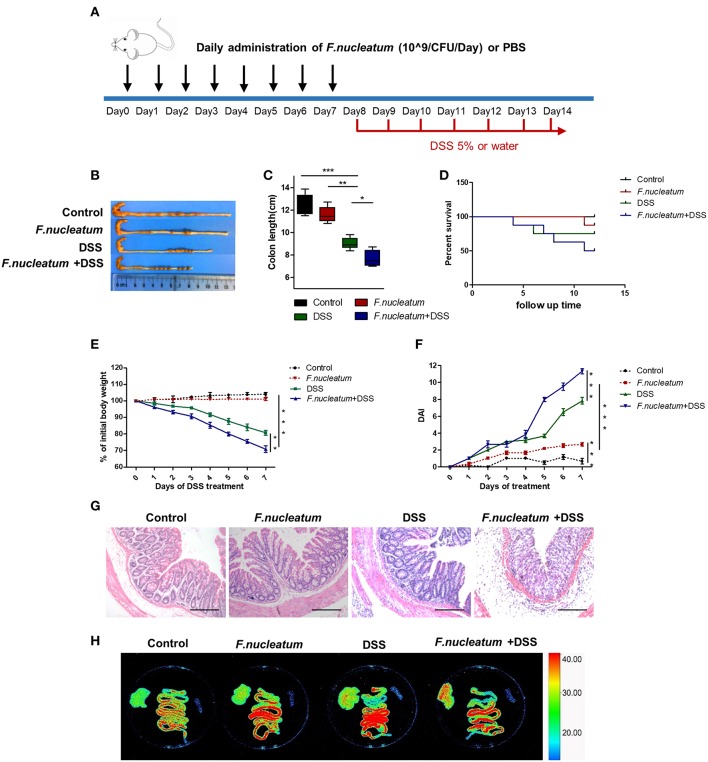
*F. nucleatum* increased the susceptibility of mice to DSS-induced experimental colitis. BABL/C mice were subjected to a DSS-induced colitis induction protocol using 5% DSS in drinking water for 7 consecutive days. **(A)** *F. nucleatum* and DSS treatment schedule. **(B,C)** Macroscopic appearance and quantification of colon length was determined at day 7 after DSS induction of colitis, *n* = 6 mice per group. Representative images are shown. **(D)** Survival curves of mice in this system, *n* = 6 mice per group. **(E)** Body weights determined daily for all mice, *n* = 6 mice per group. **(F)** Daily DAI scores for all mice, *n* = 6 mice per group. **(G)** Serial sections of colon tissues were stained with H&E to investigate tissue damage. Representative images are shown. Scale bars, 50 μm. **(H)** *Ex vivo* images of the liver, spleen, and intestine 12 h after gavage of mice with ERFP-*E.coli*. Representative images are shown. Each experiment was performed at least three times. Data are presented as means ± SD. ^*^P < 0.05; ^**^P < 0.01; ^***^P < 0.001; Analysis of Variance (ANOVA) and Student's *t*-test (two-tailed).

To assess if administering *F. nucleatum* would alter the course of UC progression, we conducted studies with the DSS-induced colitis mouse model. Mice were gavaged daily for 7 days with *F. nucleatum* prior to induction of colitis using DSS (Figure 2A). Pretreatment of mice with *F. nucleatum* was associated with increased loss of colon length and body weight in response to DSS administration, with *F. nucleatum*-treated mice having a higher disease activity index (DAI) score (Figures 2B–F). *F. nucleatum* also increased mucosal necrosis and inflammatory cell infiltration upon DSS treatment, leading to lower mouse survival rates (Figures 2D,G). Intestinal permeability was also increased in this context, as evidenced by increased liver translocation of genetically engineered *E.coli* expressing enhanced red fluorescent protein (ERFP-*E.coli*) in the mice that had been gavaged with *F. nucleatum* (Figure 2H).

### *F. nucleatum* Induces M1 Macrophage Infiltration and Activation in Colitic Mice

**Figure 3:**
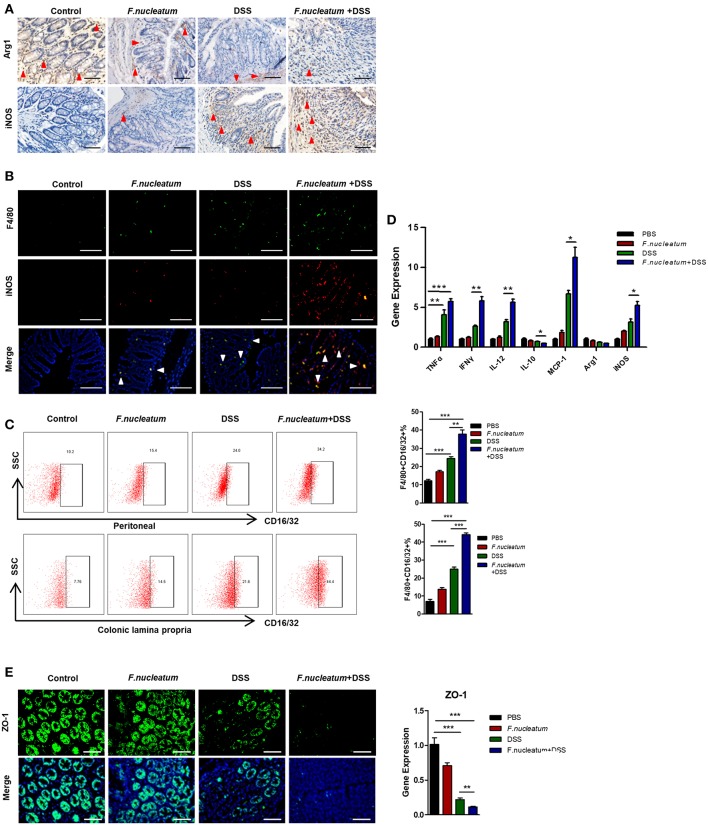
*F. nucleatum* induces M1 macrophage infiltration and activation in colitic mice. **(A)** Representative images of colon Arg1 and iNOS levels measured by IHC. Scale bars, 25 μm. **(B)** Sections of colon tissues were immunostained with F4/80 (green) and iNOS (red) macrophages; DAPI (blue) served to stain nuclei. Representative images are shown. Scale bars, 50 μm. **(C)** Peritoneal and LP cells were stained with F4/80 and CD16/32 and assessed by flow cytometry. The ratios of F4/80 and CD16/32 positive cells are shown. **(D)** TNF-α, IFN -γ, IL-12, IL-10, MCP-1, Arg-1, iNOS, and ZO-1 were measured by qRT-PCR in colon tissue samples. **(E)** Sections of colon tissues were immunostained with ZO-1 (green) and DAPI (blue) from each group. Representative images are shown. Scale bars, 10 μm. Each experiment was performed at least three times. Data are presented as means ± SD. ^*^P < 0.05; ^**^P < 0.01; ^***^P < 0.001; Analysis of Variance (ANOVA) and Student's t-test (two-tailed).

We next investigated proinflammatory macrophages in this murine model system. *F. nucleatum* feeding was linked with increased M1 macrophage levels and decreased M2 macrophage levels in sections of intestinal tissues, based on staining for the respective M1 (inducible nitric oxide synthase, iNOS) and M2 (arginase1, Arg1) markers (Figure 3A). Immunofluorescence showed that iNOS+F4/80+ double positive cells were highest in the *F. nucleatum* + DSS mice group compared to other groups, which suggested a remarkable increase of M1 macrophages infiltration after *F. nucleatum* treatment in the DSS-induced model (Figure 3B). We next isolated cells from the peritoneum and lamina propria (LP) and confirmed that *F. nucleatum* gavage was associated with increased proinflammatory M1 cell levels in both of these sites upon DSS treatment (Figure 3C). Levels of M1 markers and cytokines including TNF-α, IFN-γ, IL-12, MCP-1, and iNOS, were all up-regulated in mice of the *F. nucleatum* + DSS group, while levels of the M2 markers IL-10 and Arg1 were markedly down-regulated (Figure 3D). We also measured levels of ZO-1, which is a key tight junction protein linked with barrier integrity maintenance (29). Levels of ZO-1 were significantly lower in mice that were fed *F. nucleatum* and administered DSS relative to controls (Figures 3D,E). There is known to be cross-talk between macrophages and intestinal epithelial cells (11, 29), so these changes in M1 macrophage activation could be directly linked with altered barrier integrity and consequent UC progression.

### *F. nucleatum* Accelerates IFN-γ Activated M1 Macrophage Polarization

**Figure 4:**
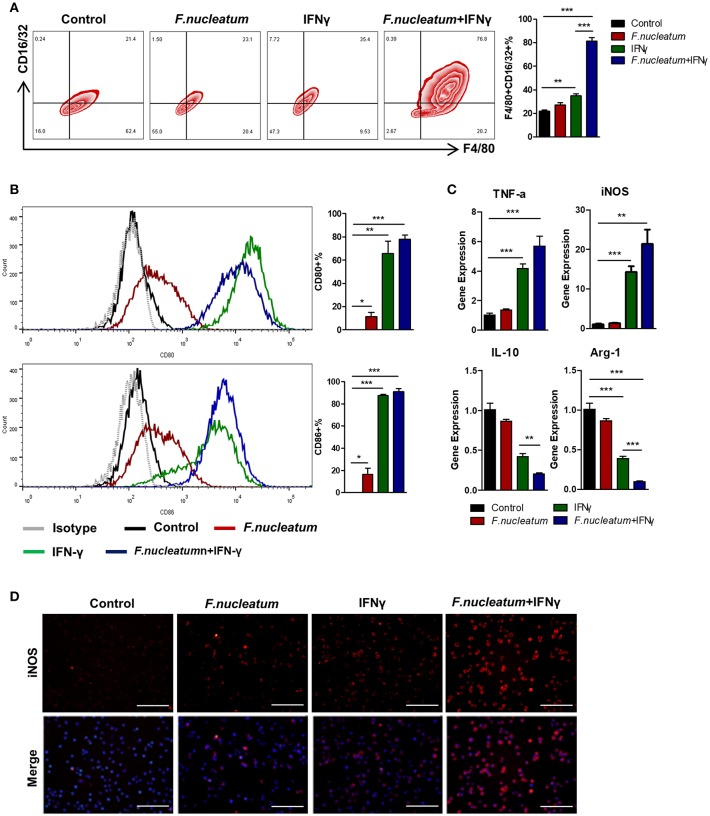
*F. nucleatum* accelerates M1 macrophage polarization. **(A)** Flow cytometry staining of BMDMs for F4/80 and CD16/32. The frequency of cells positive for these markers was determined. **(B)** BMDMs surface molecular CD80 and CD86 expression in response to bacterial and/or IFN-γ treatment. **(C)** M1 markers: TNF-α and iNOS; M2 markers: IL-10 and Arg-1, as detected by qRT-PCR. **(D)** BMDMs were immunostained with iNOS (red) and DAPI (blue) from each group. Representative images are shown. Scale bars, 50 μm. Each experiment was performed at least three times. Data are presented as means ± SD. ^*^P < 0.05; ^**^P < 0.01; ^***^P < 0.001; Analysis of Variance (ANOVA) and Student's *t*-test (two-tailed).

Changes in macrophage polarization can be driven by local host- or microbe-derived factors, potentially leading to deleterious inflammatory activation in response to locally derived LPS or IFN-γ (5, 10). As macrophages express the appropriate Toll-like receptors (TLRs) necessary to respond to Gram-negative *F. nucleatum* (30, 31), which is known to be a pro-inflammatory bacteria, we decided to further investigate the link between *F. nucleatum* and M1 macrophage polarization. We therefore generated murine bone marrow-derived macrophages (BMDMs), which were grown in the presence of *F. nucleatum* and with or without IFN-γ. *F. nucleatum* alone failed to significantly alter BMDM morphology, however in response to IFN-γ these cells exhibited increased stellate cone-shaped cells. We therefore focused on the combined effects of *F. nucleatum* and IFN-γ-induced changes in the BMDMs. IFN-γ promoted M1 macrophage expansion as measured by flow cytometry, while *F. nucleatum* alone again failed to have any effect. Importantly, however, synergy between IFN-γ and *F. nucleatum* was observed, leading to enhanced macrophage polarization (Figure 4A). Expression of CD80 and CD86, which are T cell co-stimulatory markers, was also increased upon combined bacteria and IFN-γ treatment, but there was no significant difference between this combined treatment group and the IFN-γ alone treatment group (Figure 4B). This result may lead to the fact that macrophages induced by *F. nucleatum* could not further stimulate the proliferation and differentiation of T lymphocytes. At the mRNA level, *F. nucleatum* alone did not alter expression of M1 or M2 markers obviously, but the common treatment of *F. nucleatum* and IFN-γ synergistically reduced M2 markers, though it did not increase M1 markers significantly when compared to IFN-γ alone treatment, which may originate from the inconsistency of changes in M1 and M2 macrophages (Figure 4C). In addition, immunofluorescence results of iNOS further confirmed the synergistical effect of *F. nucleatum* and IFN-γ (Figure 4D).

### *F. nucleatum* Promotes M1 Polarization via AKT2 Signaling

**Figure 5:**
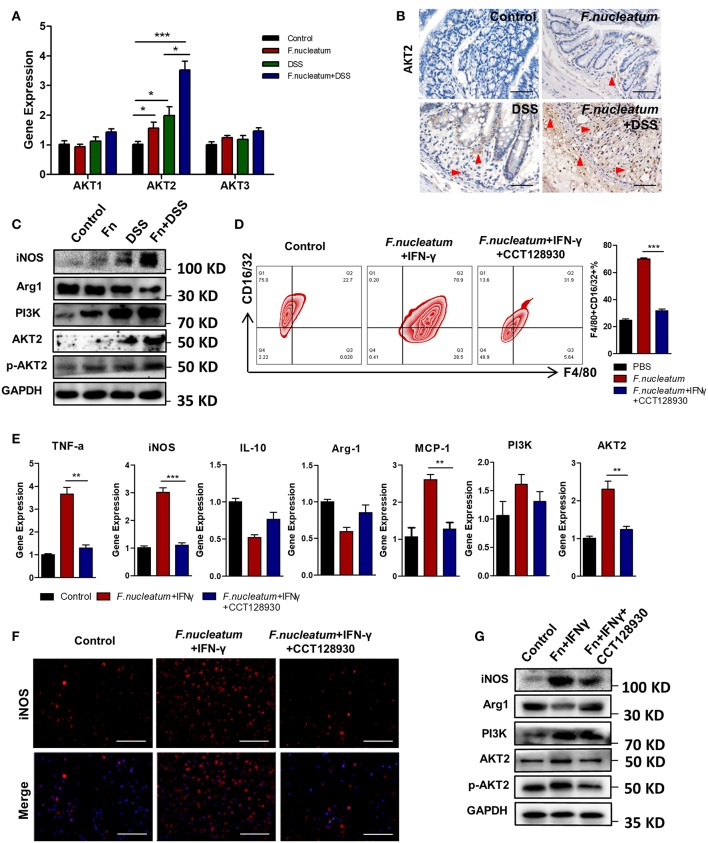
*F. nucleatum* promotes M1 polarization via AKT2 signaling. **(A)** AKT1, AKT2, and AKT3 expression measured by qRT-PCR. **(B)** Representative colon images of AKT2 IHC staining. Representative images are shown. Scale bars, 25 μm. **(C)** iNOS, Arg1, PI3K, AKT2, phospho-AKT2, and GAPDH levels measured by Western blot. **(D)** BMDMs F4/80 and CD16/32 staining by flow cytometry. Ratio of F4/80 and CD16/32 double positive cells were performed. **(E)** M1 markers, TNF-α and iNOS; M2 markers, IL-10; and Arg-1, as detected by qRT-PCR. **(F)** BMDMs were immunostained with iNOS (red) and DAPI (blue) from each group. Representative images are shown. Scale bars, 50 μm. **(G)** iNOS, Arg1, PI3K, AKT2, phospho-AKT2, and GAPDH as measured by Western blot. Each experiment was performed at least three times. Data are presented as means ± SD. ^*^P < 0.05; ^**^P < 0.01; ^***^P < 0.001; Analysis of Variance (ANOVA) and Student's *t*-test (two-tailed).

The AKT pathway has been shown to be a key regulator of macrophage polarity in many contexts (32). We therefore assessed AKT1, AKT2, and AKT3 expression in macrophages in our experimental system. We found that LP macrophages from *F. nucleatum* + DSS mice exhibited higher AKT2 protein and mRNA expression relative to DSS only mice. AKT1 and AKT3 levels were not significantly altered (Figures 5A–C). Consistent with this finding, we found that *F. nucleatum* induced only AKT2 in BMDMs (Figures 5D–G). To determine whether the M1 macrophage differentiation observed in response to *F. nucleatum* was dependent on AKT2, CCT128930 [100 μM]—which selectively inhibits AKT2—was used to treat BMDMs(Figure 5G). We saw no alterations in M1 macrophage frequency if *F. nucleatum*-treated cells were first treated with this AKT2 inhibitor (Figure 5D), suggesting that AKT2 inhibition prevents *F. nucleatum*-mediated M1 polarization. Similarly, upon AKT2 inhibition *F. nucleatum* failed to increase expression of TNF-α, MCP-1, or iNOS (Figure 5E). This confirmed the model in which *F. nucleatum* drives the M1 polarization of macrophages via the AKT2 pathway.

### Adoptive Transfer of *F. nucleatum*-Induced Macrophages Aggravates Colitis Progression

**Figure 6:**
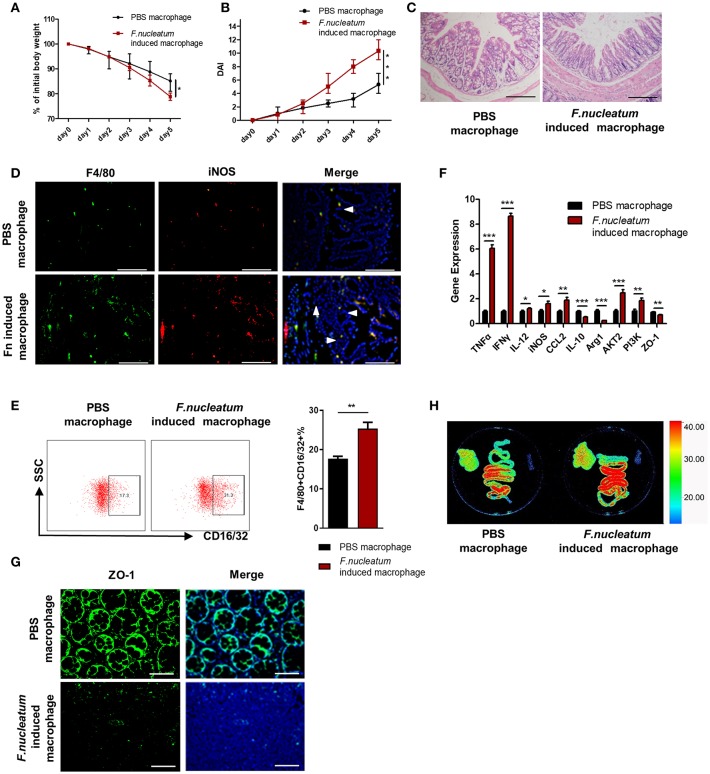
Adoptive transfer of *F. nucleatum*-induced macrophages aggravates colitis progression. *F. nucleatum*-treated BMDMs were collected and injected into the abdominal cavity of mice 48 h before initiation of DSS. **(A)** Body weight was measured daily, *n* = 6 mice per group. **(B)** DAI scores were recorded daily, *n* = 6 mice per group. **(C)** Mucosal tissues collected on day 5 were H&E stained, with representative images shown to assess colitis severity. Representative images are shown. Scale bars, 50 μm. **(D)** Mucosal tissues were stained by F4/80 (green) and iNOS (red) in macrophages; DAPI (blue) served as a nuclear stain. Representative images are shown. Scale bars, 50 μm. **(E)** F4/80 and CD16/32 staining of LP cells. Ratio of F4/80 and CD16/32 double positive cells were performed. **(F)** Expression of TNF-α, IFN -γ, IL-12, IL-10, MCP-1, Arg-1, iNOS, and ZO-1 was measured by qRT-PCR in colon tissues. **(G)** Mucosal tissues were stained with ZO-1 (green); DAPI (blue) served as a nuclear stain. Representative images are shown. Scale bars, 10 μm. **(H)** *Ex vivo* images of the liver, spleen and intestine 12 h after ERFP-*E. coli* gavage. Representative images are shown. Each experiment was performed at least three times. Data are presented as means ± SD. ^*^P < 0.05; ^**^P < 0.01; ^***^P < 0.001; Analysis of Variance (ANOVA) and Student's *t*-test (two-tailed).

In order to directly determine the physiological relevance of *F. nucleatum*-activated macrophages in the context of UC, we adoptively transferred *in vitro F. nucleatum*-induced BMDMs into mice 48 h before initiating DSS treatment, and M1 macrophages in the colon and integrity of intestinal epithelial barrier were detected. This transfer of *F. nucleatum*-induced BMDMs increased significantly the susceptibility of mice to DSS-induced experimental colitis, which was manifested in the obvious increase of weight loss, crypts destruction and inflammatory cell infiltration (Figures 6A–C), especially the skewing of M1 macrophages in the colon (Figures 6D,E). Additionally, pro-inflammatory cytokines such as TNF-α, IFN-γ, and IL-12 expression were also substantially increased in the intestine, while anti-inflammatory IL-10 was decreased (Figure 6F). We also observed increased intestinal permeability in mice that received these *F. nucleatum*-activated macrophages, as evidenced by increased liver translocation of ERFP-*E.coli* from intestine (Figure 6H) and reduced expression of ZO-1 in colon tissues (Figure 6G).

## Discussion

In the present study, we for the first time revealed the molecular mechanisms by which *F. nucleatum* aggravates the progression of UC. Through it is proinflammatory nature, *F. nucleatum* is able to induce the mobilization of macrophages and is thereby able to promote their skewing toward an M1 phenotype via the AKT2 signaling pathway. Subsequently, strong inflammatory cytokine release further damages the intestinal mucosal barrier, potentially contributing to the development and progression of UC. This report lends further support to models in which macrophages are the key players regulating mucosal barrier defenses, and further suggests that the targeting of *F. nucleatum* or AKT2 may be a viable approach to reduce or eliminate intestinal inflammation and barrier disruptions in the context of disease.

Macrophages are well known to be abundant within the intestine, where they are able to survey the luminal environment and respond to threats with the goal of maintaining and restoring local tissue homeostasis (2, 5). Numerous studies have found that the specific intestinal bacterial microbiome can determine what macrophage-mediated immune responses are induced. Exactly why certain commensal or pathogenic bacteria are able to trigger distinct immune responses, and what sets the threshold for determining whether an adaptive immune response is triggered in this environment, however, remain uncertain (33). Dysregulation of this carefully regulated distinction and of efforts to maintain local homeostasis, however, are believed to be linked with inflammatory diseases such as UC (33, 34). It has been well demonstrated that an imbalance in the intestinal microbiome is an important risk factor for the development of UC, with much emphasis being placed on the balance between probiotics and pathogens. Recent sequencing studies have confirmed that increases or decreases in certain bacteria are closely related to the occurrence and development of UC, but the exact nature of this causal relationship and the associated specific pathogenesis requires further clarification. *F. nucleatum*, an anaerobic gram-negative bacterium, has recently been identified in the oral cavity and intestines of patients with IBD, and in these individuals it exhibits an enhanced invasive ability that is not present in bacteria in healthy individuals. *F. nucleatum* has previously been shown to support colorectal tumorigenesis via the release of small peptides and short-chain fatty acids that can serve to attract myeloid-derived suppressor cells (MDSCs) to the tumor local microenvironment (27). Once present, these MDSCs can suppress immune function locally via the secretion of many factors, constraining CD4+T cell responses. Moreover, Kostic et al. have previously identified that *F. nucleatum* could shape a proinflammatory microenvironment that is conducive for intestinal tumorigenesis, and proinflammatory genes such as Ptgs2 (COX-2 mouse homolog), Scyb1 (IL-8 mouse homolog), IL-6, TNF-a, and MMP-3 were more highly expressed in intestinal tumors from mice that were treated with *F. nucleatum*, which involved the activation of NF-kB signaling. *F. nucleatum* also selectively recruited myeloid-derived immune cells but not lymphoid immune cells into colonic tumors in mice (27). These studies suggest that *F. nucleatum* may be closely related to the activities of myeloid-derived cells and activation of inflammatory signaling, despite the high degree of heterogeneity and plasticity of these infiltrated cells. In addition, since the formation of some colonic tumors was closely related to chronic inflammation, we investigated the relationship between the abundance of this bacterium and the different types of macrophages present in clinical UC samples. Our results revealed that *F. nucleatum* is present at high levels in UC patients, and its abundance correlates closely with the severity of disease and the production of cytokines including IFN-γ. Consistent with the inflammatory UC microenvironment, we also observed primarily proinflammatory M1 macrophages, but not M2 macrophages, in clinical samples (Figure 1), a finding distinct from what is observed in a tumor setting (27). Work by Christoffersen et al. has recently shown that a number of gram-negative bacteria favor M1 macrophage responses and induce cytokine secretion by these cells *in vitro* (35). Commensal *Enterococcus faecalis* has further been shown to promote comparable polarization and local DNA damage in intestinal epithelial cells (36), supporting a causative link between bacterial colonization and macrophage-induced inflammation.

Using a DSS-induced colitis model, we demonstrated that *F. nucleatum* accelerated DSS-induced mucosal inflammation, as evidenced by increased weight loss, inflammatory cell infiltration, and histopathological scores (Figure 2). Macrophages that can rapidly respond to different pathogens constitute an important part of the natural immune defenses, and the role played by different macrophage subtypes in the context of inflammation and mucosal injury thus differs significantly. In this UC model, we observed increased M1 macrophage repolarization after *F. nucleatum* treatment (Figures 3A–C). Many studies have confirmed that M1 macrophages can trigger strong local inflammation, secreting large amounts of chemokines and inflammatory factors. In our *in vivo* and *in vitro* experiments, *F. nucleatum* was able to promote the secretion of inflammatory factors such as IFN-γ, TNF-α, and IL-12, while also inhibiting the secretion of anti-inflammatory factors such as IL-10. Production of monocyte chemotactic protein-1 (MCP-1) also increased significantly (Figure 3D). MCP-1 can recruit more immune cells, especially mononuclear macrophages derived from peripheral tissues, to invade sites of inflammation, while IFN-γ and TNF-α can damage the intestinal mucosal barrier and promote bacterial translocation, as revealed by increased ERFP-labeled *E.coli* translocation in the liver (Figure 2H), coupled with decreased ZO-1 expression in the colon in our studies (Figure 3D).

In order to investigate the direct and indirect effects of *F. nucleatum* on M1 macrophages, we studied BMDMs *in vitro*. We found that BMDMs infected alone with *F. nucleatum* at a multiplicity of infection (MOI) of 100 for 24 h could only induce low levels of M1 polarization. In order to better mimic the inflammatory microenvironment of the UC model, we treated macrophages with a combination of *F. nucleatum* and IFN-γ, which revealed significantly enhanced M1 macrophage polarization compared to controls (Figure 4). Thus, our data confirmed the synergistic effect of these two signals *in vitro*, suggesting that the inflamed intestinal microenvironment may provide the essential conditions for *F. nucleatum*-induced M1 macrophage repolarization. To clarify the source of M1 macrophages, we also investigated M2 polarization in this same model system, observing a substantial decrease in these cells in colitic mice relative to controls after *F. nucleatum* treatment, as evidenced by their specific marker Arg1 and their characteristic secretion of IL-10 (Figures 3A,D, 5C). Consequently, we can conclude that *F. nucleatum* can induce intestinal macrophages to polarize into an M1 phenotype while inhibiting their M2 polarization, further confirming macrophage plasticity. Recent work has suggested that *F. nucleatum* can favor TLR4-mediated M2 polarization within colorectal tumors (37), suggesting that the immune response to these bacteria is context-dependent and heterogeneous, with the local environment regulating functional outcomes. Moreover, the traditional M1/M2 macrophage polarization model is also insufficient to describe the full range of macrophage activity (38), and as such further studies should be performed to more deeply explore the association between *F. nucleatum* and the functions of various macrophage subtypes both *in vivo* and *in vitro*.

Recent work has shown that a number of tightly controlled pathways regulate the inflammatory and immunosuppressive activity of particular macrophage subsets, maintaining intestinal homeostasis in most healthy individuals. Previous studies have found that *F. nucleatum* can readily induce robust cytokine production *in vitro* when used to stimulate many different types of cells. Responses induced by this bacterium include FAD-1-mediated beta-defensin 2 secretion from oral epithelial cells (39), Fap2 and RadD-mediated lymphocyte inactivation and death (40) and TLR4-mediated IL-6, IL-8, and TNF-α secretion (41). *F. nucleatum* can also activate natural killer cells in the context of periodontal disease (42). These past results strongly suggest that *F. nucleatum* can actively alter local inflammatory conditions in a manner that may allow this opportunistic pathogen to flourish. *In vivo, F. nucleatum* can also induce TLR4-mediated placental inflammation, leading to fetal demise (43). In addition, *F. nucleatum* in Apc^min/+^ mice may induce the expansion of myeloid cells, with subsequent inflammation activating oncogenes and Wnt genes, which support tumorigenesis (27). As TLR4 is a classical signaling pathway by which gram-negative bacteria induce innate immune responses, and the main component of their cell wall, lipopolysaccharide, is an effective agonist for TLR4, we conjectured that this may be a major mechanism by which *F. nucleatum* induces M1 macrophage polarization: TLR stimulation and Akt activation propagated by the downstream NF-κB signaling pathway, driving the activation of inflammatory genes that, when chronically active, can cause severe pathologic tissue damage via epithelial barrier integrity breakdown (44, 45). Recently, the AKT family of proteins (AKT1, AKT2, and AKT3) in particular has been highlighted as being key regulators of macrophage-induced inflammation, with AKT2 signaling regulating M1 macrophage activation and IBD pathogenesis (32, 46). We found that AKT2, but not AKT1 or AKT3, was also expressed by intestinal macrophages *in vivo*, with elevated expression during inflammation that was further increased by *F. nucleatum* treatment (Figures 5A–C). Differentiated BMDMs *in vitro* also exhibited elevated AKT2 expression upon *F. nucleatum* stimulation, and AKT2 inhibition with *CCT128930* largely ablated the polarization of M1 macrophages and the secretion of cytokines. (Figures 5D–G). Together, these findings reveal a new mechanism by which AKT2 signaling regulates M1 macrophage repolarization in colitic mice following *F. nucleatum* treatment, and targeting this pathway with a specific inhibitor could therefore help to resolve chronic intestinal inflammation.

**Figure 7:**
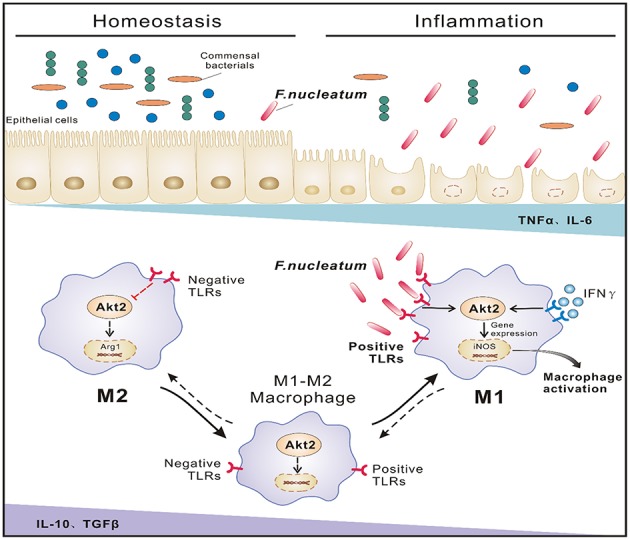
Proposed model of *F. nucleatum*-induced M1 macrophage polarization and proinflammatory cytokine production via the AKT2 pathway in the context of UC. In the inflammatory state of UC, increased *F. nucleatum* induces mononuclear macrophage infiltration by activating AKT2 signaling and differentiation into iNOS-positive M1 cells, secreting a large amount of inflammatory factor TNF-α and IFN-γ, etc., which in turn damages the intestinal mucosal epithelial barrier and increases intestinal permeability and bacterial translocation, leading to increased inflammation and progression of the disease.

In conclusion, our data demonstrate that in the inflammatory context of UC, increased *F. nucleatum* abundance induces iNOS-positive M1 macrophage polarization by activating AKT2 signaling in the colon, and these activated macrophages can secrete large quantities of inflammatory factors such as TNF-α, IFN-γ, and MCP-1, which in turn recruit more macrophages into sites of inflammation, damage the mucosal barrier, promote bacterial translocation and eventually lead to increased disease progression (Figure 7). In the future, we will focus on how to prevent the progression and deterioration of UC by interfering with the growth of *F. nucleatum* and its downstream signals, thus providing new strategies for the treatment of UC.

## Materials and Methods

### Patient Samples

Colon tissues, blood and fecal specimens from 30 UC patients and 16 healthy controls following endoscopic examination from the Department of Gastroenterology, Nanfang Hospital from 2014 to 2016 were collected. Standard endoscopy was used for official UC diagnosis along with appropriate histology and clinical scoring. Mayo scores were determined in line with previously described methods. Any patients that were pregnant, had evidence of cancer or short bowel syndrome, or sepsis were not included in the present study. The baseline characteristics of the patients are listed in Supplementary Table 1. All aspects of this research were conducted consistently with institutional ethical guidelines, all subjects have signed an informed consent form, and the study was approved by the Southern Medical University medical ethics committee (Number: NFEC-2014-035).

### Bacterial Strains and Growth Conditions

*F. nucleatum* strains were isolated from the feces of a UC patient via selective medium using stool samples collected in an approved manner, in line with Institutional Review Board guidelines. A Gram staining kit was utilized for *F. nucleatum* identification, with confirmation via 16S ribosomal DNA sequencing. *F. nucleatum* was grown using brain-heart infusion broth containing yeast extract (5 mg/mL), hemin (5 mg/mL) and menadione (1 mg/mL) at 37°C (10% H2, 5% CO2, and 85% N2) with an AnoxomatTM MarkII anaerobic system (Mart Microbiology).

### Animals

Six- to eight-week-old male BABL/c mice were from the Laboratory Animal Center of Southern Medical University and were housed in standard specific pathogen-free conditions. The Institutional Animal Care and Use Committee of Nanfang Hospital, Southern Medical University, China approved this study and all animal protocols were performed in accordance with their recommendations.

### Acute Colitis Induction and Bacterial Treatment

Mice were randomly divided into 4 groups: Control, *F. nucleatum* treated only (*F. nucleatum*), DSS (40 kDa, Millipore Corporation, Billerica, MA, USA) treated only (DSS) and *F. nucleatum*+DSS-treated (*F. nucleatum*+DSS). Mice receiving bacteria were subject to a 1-week period of daily gavage with 500 ul bacterial solution (10^9^ CFU/ml) in PBS, while control mice received only PBS. One week after this infection, colitis was induced in appropriate groups via water-mediated application of 5% (wt/vol) DSS for 7 days. DSS was prepared fresh every 2 days. Mean cage DSS consumption was recorded daily. Control mice received normal drinking water. Mice in the DSS group were monitored for weight loss, stool consistency and bleeding for days 0 to 7 of DSS treatment. Stool blood was measured via Hemoccult II test (Beckman Coulter, Oakville, ON, Canada). On day 7, mice were sacrificed.

For adoptive transfer experiments, treated BMDMs were harvested and resuspended at 5 × 10^5^ cells in 30 μl DMEM and were transferred into mice 48 h prior to DSS initiation by intraabdominal injection. Weight loss, stool consistency and bleeding were monitored for 5 days following DSS administration, as above, and mice were then sacrificed.

### Clinical Colitis Scoring

Colitic disease activity index (DAI) was calculated according to stool consistency, fecal blood and weight loss. For weight loss: none = 0, 1–5% = 1, 5–10% = 2, 10–20% = 3, and ≥20% = 4; stool consistency: 0 = well-formed pellets, soft but formed = 1; very soft = 2; diarrhea = 3; and fecal blood: 0 = no blood, 1 = positive hemoccult, 2 = visible blood in stool, 3 = gross rectal bleeding. Scores were averaged to determine the final DAI score.

### Histopathology

Paraffin-embedded colon sections 4 μm thick were H&E stained, and then a blinded pathologist analyzed them. On the basis of Geboes criteria, the severity of UC was graded from 0 to 5 (47). Histopathological results were determined on the basis of the extent of inflammation, ulceration and epithelial damage as described previously.

### Peritoneal Macrophage Collection

Injection of 4 ml of RPMI-1640 containing 5% FBS and 0.5 mM ethylene diamine tetraacetic acid (EDTA) into the peritoneum was used for peritoneal macrophage collection. Some cells were immediately used to measure M1 macrophage frequency, while the remaining cells were isolated following 10 minutes of centrifugation at 300 × g and were cultured at 37°C in complete RPMI-1640 media. Following a 2-h incubation, non-adherent cells were removed and adherent macrophages were grown for longer periods in complete media as appropriate.

### Lamina Propria Cell Isolation

Dissected colons were washed in penicillin/streptomycin-supplemented PBS and minced, followed by incubation in a solution of RPMI-1640 (Gibco, Grand Island, NY, USA) with 3% FBS (Gibco, Grand Island, NY, USA), 0.5 mM dithiothreitol (Sigma-Aldrich), 5 mM EDTA (Sigma) and antibiotics at 37°C for 30 min in order to eliminate the epithelial layer. Tissue was then incubated at 37°C in RPMI-1640 containing 0.5% Collagenase D (Roche) and 0.05% DNase (Roche) for 30 min and shaken gently. Derived cells were then formed into a single cell suspension, filtered via a 70 μm strainer and isolated for flow cytometry.

### *In vitro* Generation and Polarization of BMDMs

Bone marrow-derived macrophage cells from male BALB/c mice were collected via flushing the femur and tibia with PBS. These BMDMs were resuspended in complete RPMI-1640 containing M-CSF (100 ng/mL, Peprotech, USA). After 7 days at 37°C, with media being changed once, cells were harvested by scraping and grown in a 24-well plate at 5 × 10^5^ cells/well overnight. To induce polarization, BMDMs were treated with *F. nucleatum* for 24 h and with IFN-γ (20 ng/ml, Peprotech, USA) for 2 h before *F. nucleatum* treatment. For this treatment, *F. nucleatum* colonies from the plate were resuspended in antibiotic-free RPMI-1640 medium at 5 × 10^6^ CFU/ml. A total of 2 mL of this bacterial solution was used for BMDM infection.

### Flow Cytometry

Peritoneal macrophages, lamina propria cells, and BMDMs were washed and stained for 30 min at 4°C using FITC rat anti-mouse F4/80 (BD Bioscience, USA) and PE anti-mouse CD16/32 (BD Bioscience, USA). F4/80 served as a macrophage marker, with F4/80+CD16/32+ cells considered to be M1 macrophages. A FACSCanto II cytometer (BD) was used to collect the data, which were analyzed with the FACSDiva (BD) and FlowJo software packages (TreeStar, USA).

### Immunohistochemistry

Paraffin-embedded tissue sections (4 μm) underwent appropriate heat-induced antigen retrieval according to standard protocols. Samples were then treated with 3% hydrogen peroxide for 10 min, blocked and incubated overnight at 4°C with murine anti-*F. nucleatum* (1:10, prepared by our laboratory bacterial immunized mice), rabbit anti-Arg1 (Proteintech, Wuhan, China), rabbit anti-iNOS (Proteintech, Wuhan, China), rat anti-F4/80 (Proteintech, Wuhan, China), rabbit anti-PI3K (Abcam, Cambridge, MA, USA) and mouse anti-AKT2 (Abcam, Cambridge, MA, USA). At the same time, we set up a control by selecting serum from the same animal of the first antibody instead of the first antibody in each group of experiments. The next day samples were washed prior to incubation with secondary antibodies against mouse or rabbit IgG conjugated to HRP (Zhongshang Goldenbridge), and a 5–10 min incubation with 3,3′-diaminobenzidine tetrachloride was used to visualize staining via light microscopy (Olympus, Japan).

### Immunofluorescence

Rehydrated tissue samples were generated as above, blocked for 1 h, and incubated overnight at 4°C with FITC- and PE-labeled antibodies against F4/80 and iNOS (BD Bioscience, USA) or FITC-labeled antibodies against ZO-1 (Life Technologies, USA). Tissues were then washed, stained with DAPI for 10 min, and imaged via fluorescent microscopy (Olympus, Japan).

### Western Blotting

RIPA (Vazyme, Nanjing, China) buffer supplemented with PMSF was used to lyse tissue samples, and total protein content was measured via BCA assay (Merck, Darmstadt, Germany). All protein was run on a 10% SDS-PAGE gel prior to transfer to a PVDF membrane (Bio-Rad, Marnes-la-Coquette, France). This blot was then blocked with 5% non-fat milk prior to incubation overnight at 4°C with appropriate primary antibodies, which were as follows: rabbit anti-Arg1 (Proteintech,Wuhan, China), rabbit anti-iNOS (Proteintech, Wuhan, China), rabbit anti-PI3K (Abcam, Cambridge, MA, USA), rabbit anti-p-AKT2 (Abcam, Cambridge, MA, USA) and rabbit anti-AKT2 (Abcam, Cambridge, MA, USA), and rabbit anti-GAPDH (ZSGB-BIO, Beijing, China). At the same time, we set up a control by rabbit IgG (Proteintech, Wuhan, China) instead of the first antibody in each group of experiments. Blots were then washed and probed with secondary anti-rabbit IgG conjugated to HRP prior to ECL detection and visualization.

### *Ex vivo* Imaging

Mice were administrated with 500 ul ERFP-labeled genetic engineering *E.coli* solution (10^9^ CFU/ml) in PBS and were euthanized after 12 h, and liver, spleen and intestine were collected. Bruker Small Animal Live Imaging System was used to assess PE signal in these tissues, with radiant efficiency assessed via Bruker MI SE software.

### Quantitative Real-Time PCR

TRIzol (Thermo Fisher Scientific) was used to isolate total cellular RNA, which was then reverse transcribed using the PrimeScript Kit (TaKaRa). Real-time quantitative reverse transcription PCR (qRT-PCR) was then performed with a SYBR mix (TaKaRa) via the following cycling: 95°C for 30 s, then 40 cycles of 95°C for 5 s, 55°C for 30 s, and 72°C for 30 s. A Roche LightCycle^@^ 480II system was used for this reaction. The changes in expression were determined via 2^−ΔΔ*CT*^ calculations. Primer sequences are shown in Supplementary Table 2.

### Quantitative Real-Time PCR for *F. nucleatum*

The bacterial DNA of clinical stool samples was extracted using the Tiangen Genomic DNA Extraction Kit (Qiagen, Hilden, Germany). 16s primers for *F. nucleatum* quantification were F-TGCGATAAGCCTAGATAAGTTGCA;R-CTTAATAGATTGCTCCATTCGGAAA. Primers for total bacterial DNA were F-ACTCCTACGGGAGGCAGCAGT; R-GTATTACCGCGGCTGCTGGCAC. Each primer pair was used for PCR, with the products cloned into a pUCm-T vector (Sangon, Shanghai, China) to determine standard copy numbers. qRT-PCR was performed similarly to as above, with a modified cycle of 95°C for 5 min, then 45 cycles of 95°C for 10 s, 60°C for 20 s, and 72°C for 20 s. The standard curve generated using plasmid DNA was used to determine copy number, with *F. nucleatum* levels determined according to the following formula: log10 (*F. nucleatum* copies/total bacterial copies).

## Statistical Analysis

Each experiment was independently conducted three times, with data expressed as mean ± SEM. A two-tailed Student's *t*-test was used to compare pairs of groups, with one-way ANOVAs used for multiple groups using Dunnett's *post-hoc* comparisons. SPSS 19.0 (IBM) was used for all statistics, with the threshold of significance being *p* < 0.05.

## Ethics Statement

### Patient Samples

Colon tissues, blood, and fecal specimens from 30 UC patients and 16 healthy controls following endoscopic examination from the Department of Gastroenterology, Nanfang Hospital from 2014 to 2016 were collected. Standard endoscopy was used for official UC diagnosis, along with appropriate histology and clinical scoring. Mayo scores were determined in line with previously described methods. Any patients that were pregnant, had evidence of cancer or short bowel syndrome, or sepsis were not included in the present study. All aspects of this research were conducted consistently with institutional ethical guidelines, all subjects have signed an informed consent form, and the study was approved by the Southern Medical University medical ethics committee (number: NFEC-2014-035).

### Animals

6–8 week old male BABL/c mice were from the Laboratory Animal Center of Southern Medical University, and were housed in standard specific pathogen free conditions. The Institutional Animal Care and Use Committee of Nanfang Hospital, Southern Medical University, China approved this study and all animal protocols were performed in accordance with their recommendations.

## Author Contributions

LL was responsible for performing experiments, analyzed the data, and wrote the paper. LpL was responsible for performing experiments and helped analyze the data. HL collected, organized data and assisted with some experiments. MW collected clinical specimens, recorded clinical data and assisted with modification of the manuscript. BL provided important help in experimental operation and assisted with some experiments. MX collected clinical specimens, recorded clinical data and assisted with some experiments. JD assisted with some experiments. YC was responsible for experimental design, guidance, and revising the paper.

### Conflict of Interest Statement

The authors declare that the research was conducted in the absence of any commercial or financial relationships that could be construed as a potential conflict of interest.
